# Simultaneous visualisation of calcified bone microstructure and intracortical vasculature using synchrotron X-ray phase contrast-enhanced tomography

**DOI:** 10.1038/s41598-017-13632-5

**Published:** 2017-10-16

**Authors:** Juan A. Núñez, Alice Goring, Eric Hesse, Philipp J. Thurner, Philipp Schneider, Claire E. Clarkin

**Affiliations:** 1 Biological Sciences, Faculty of Natural and Environmental Sciences, University of Southampton, SO17 1BJ England, UK; 2 Bioengineering Sciences Research Group, Faculty of Engineering and the Environment, University of Southampton, SO17 1BJ England, UK; 3 Heisenberg-Group for Molecular Skeletal Biology, Department of Trauma, Hand and Reconstructive Surgery, University Medical Center Hamburg-Eppendorf, Martinistrasse 52, D-20246 Hamburg, Germany; 4 Department of Anatomy and Cell Biology, Indiana University School of Medicine, Indianapolis, IN USA; 5 Institute of Lightweight Design and Structural Biomechanics, Vienna University of Technology, 1060 Vienna, Austria

## Abstract

3D imaging of the bone vasculature is of key importance in the understanding of skeletal disease. As blood vessels in bone are deeply encased in the calcified matrix, imaging techniques that are applicable to soft tissues are generally difficult or impossible to apply to the skeleton. While canals in cortical bone can readily be identified and characterised in X-ray computed tomographic data in 3D, the soft tissue comprising blood vessels that are putatively contained within the canal structures does not provide sufficient image contrast necessary for image segmentation. Here, we report an approach that allows for rapid, simultaneous visualisation of calcified bone tissue and the vasculature within the calcified bone matrix. Using synchrotron X-ray phase contrast-enhanced tomography we show exemplar data with intracortical capillaries uncovered at sub-micrometre level without the need for any staining or contrast agent. Using the tibiofibular junction of 15 week-old C57BL/6 mice post mortem, we show the bone cortical porosity simultaneously along with the soft tissue comprising the vasculature. Validation with histology confirms that we can resolve individual capillaries. This imaging approach could be easily applied to other skeletal sites and transgenic models, and could improve our understanding of the role the vasculature plays in bone disease.

## Introduction

Today, the role of the vasculature in skeletal pathology remains poorly understood as the structural nature of skeletal tissue has made it exceptionally difficult to directly investigate the vasculature within it. Bone blood vessels are deeply encased in the calcified tissue and techniques applicable to soft tissue are frequently difficult to apply to the skeleton, such as confocal laser scanning microscopy or light sheet microscopy^1,2^. What we do know is that the processes of bone growth, mineralisation, repair and rejuvenation are dependent upon the vascular supply^3–5^. Bone cells can communicate with the vasculature^6–9^ in normal conditions and a loss of this communication could be involved in the onset of bone pathologies. Indeed, loss of osteoblast derived pro-angiogenic vascular endothelial growth factor (VEGF) in mice results in an osteoporotic phenotype and also negatively impacts fracture repair^6,10^ and reduced levels of circulating VEGF have been found in postmenopausal women^11^. Improved imaging allowing for rapid and accurate quantification of the bone vasculature could better inform our ideas about the role the vasculature plays in driving degenerative bone disease such as osteoporosis.

Micro-computed tomography (µCT) arose as an excellent 3D and non-destructive imaging technique, in addition to conventional two-dimensional (2D) examination of histological sections by light microscopy. Standard µCT or X-ray absorption-based µCT essentially maps the local X-ray attenuation of the specimen in 3D at a micrometre range. The capabilities of standard µCT can be significantly extended when synchrotron sources (SR) are used as X-ray sources compared to lab-based X-ray sources, as higher spatial resolutions can be achieved at sufficient signal-to-noise ratios and scanning times to resolve porosity present in cortical bone within a few minutes, such as the vascular canal networks and osteocyte lacunae^12–14^.

While canals in cortical bone can readily be identified and characterised in standard X-ray computed tomographic data in 3D using the negative imaging approach^15^, the soft tissue comprising blood vessels that are putatively contained within the canal networks do not provide the sufficient image contrast necessary for image segmentation. Thus to date it has not been possible to determine whether blood vessels are present within these canals without the addition of a contrast agent.

Recently, Fratini *et al*.^16^ have highlighted the advantages of phase-contrast synchrotron-based computed tomography (SR CT) to study soft tissue comprising the murine neurovascular network in a non-destructive, 3D fashion without the addition of contrast agents. Phase contrast SR CT, or more specifically propagation based phase contrast imaging, has proved to be an incredibly simple yet powerful approach to exploit the sample-induced phase shift and increased image contrast for low-absorbing samples when using synchrotron light sources, or more generally coherent light sources. By leaving an appropriate space between the sample and the detector, and through constructive and destructive interference of neighbouring and coherent X-ray waves, the phase shift of the X-ray beam caused by the sample is transformed into intensity variations that are then recorded by the detector. This arrangement allows the study of samples with either low X-ray absorption or multiple constituents. This year alone phase contrast SR CT has been successfully applied to visualise the microstructure of a broad range of soft tissues including spindles and nerves in murine skeletal muscle^17^, myocyte orientation in cardiac tissue^18^, the vasculature of a whole heart^19^, murine spinal cord^20^, and 3D printed hybrid cartilage contructs^21^.

Our objective was to apply this methodology to the study of hard tissues, specifically the cortical bone of the tibiofibular junction of adult mice in attempt to detect the presence and size of blood vessels within the canal networks, in the absence of any contrast agent. We have now shown that propagation-based phase contrast-enhanced tomography can indeed resolve soft tissue comprising blood vessel details in undecalficied murine bone, when an appropriate propagation distance is chosen. Furthermore, this technique allows for simultaneous visualisation of the cortical bone microstructure including osteocyte lacunae and canal networks alongside the blood vessels themselves.

## Results

### Detection of intracortical blood vessels using propagation-based phase contrast-enhanced imaging

**Figure 1:**
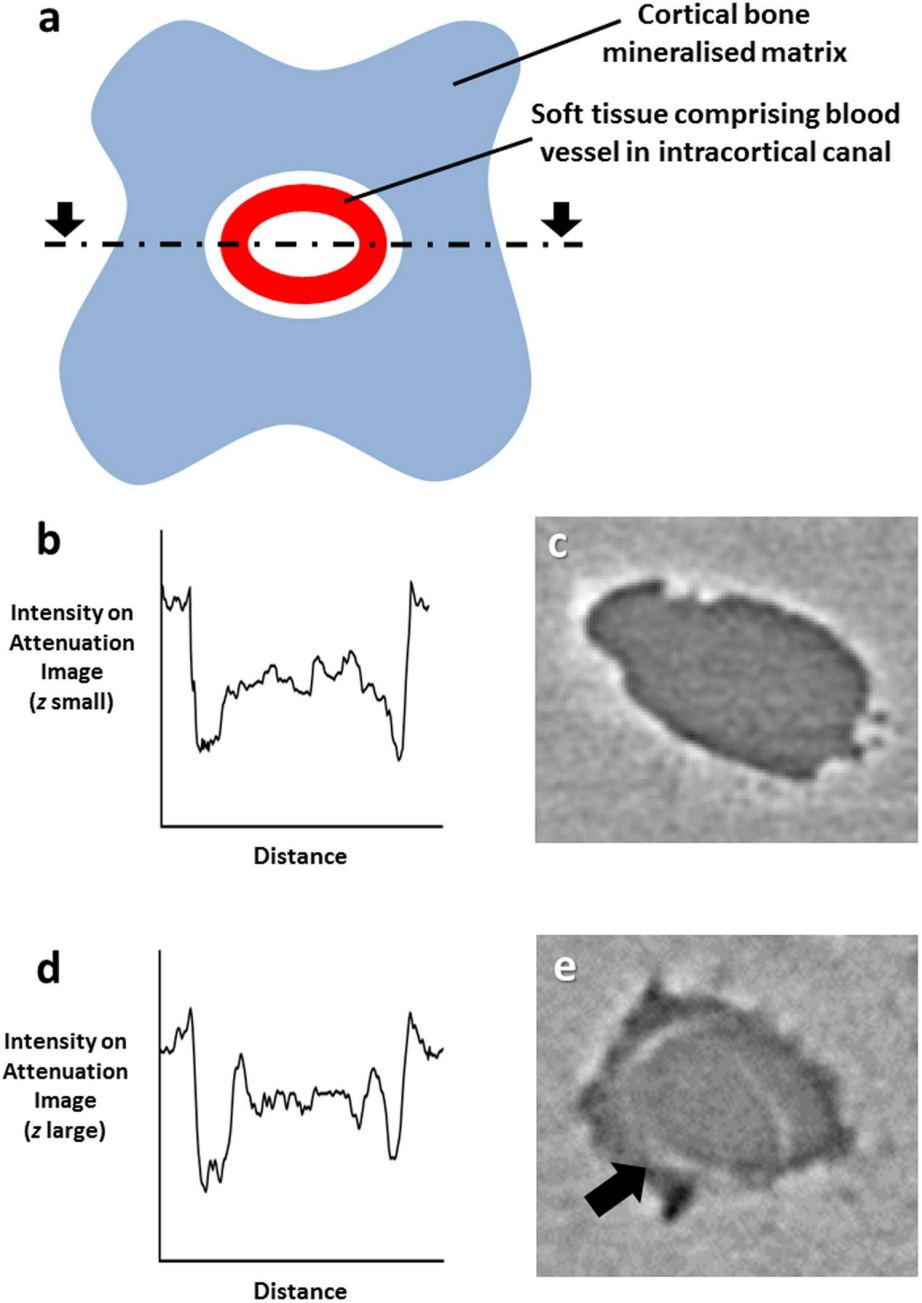
Phase contrast-enhanced visualisation of the soft tissue comprising blood vessels in calcified bone. (**a**) Schematic representation of an intracortical canal occupied by a blood vessel. Image intensity profile plotted along a line intercepting intracortical canal and internal blood vessel for standard X-ray micro-computed tomography (µCT) (**b**). Magnified area on µCT reconstructed slice containing an intracortical canal (0.325 µm voxel size, 15 mm propagation distance) (**c**). Image intensity profile plotted along a line intercepting intracortical canal and internal blood vessel for phase contrast-enhanced µCT (**d**). Magnified area on phase contrast-enhanced µCT reconstructed slice containing an intracortical canal with internal blood vessel visible (0.325 µm voxel size, 25 mm propagation distance) (**e**).

**Figure 2:**
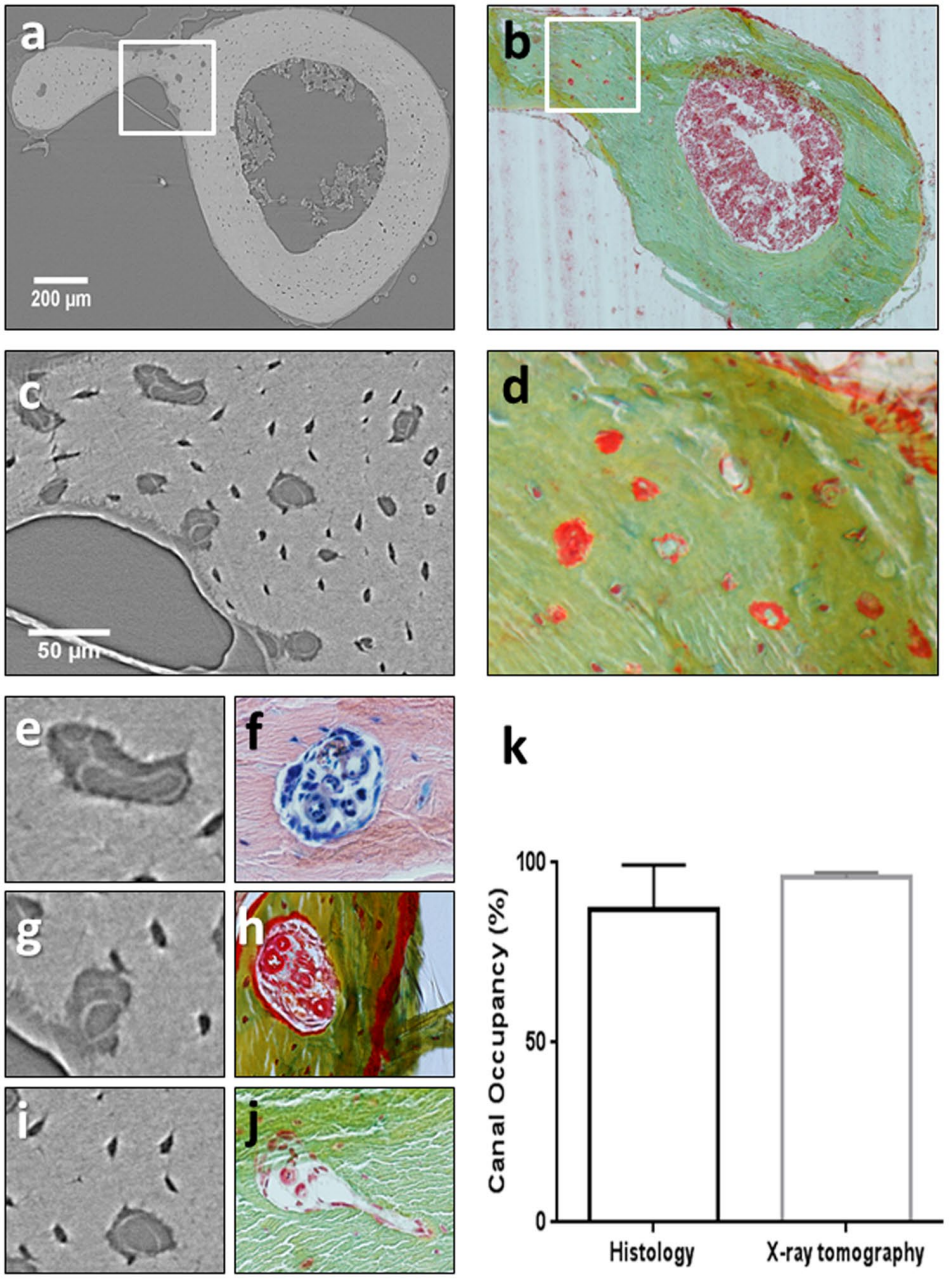
Detection of intracortical blood vessels by phase contrast-enhanced micro-computed tomography and validated by histology. (**a**) (µCT) slice of the murine tibiofibular junction with large blood vessels visible (1.3 µm voxel size). (**b**) Histological cross-section of the tibiofibular junction (Pentachrome staining). (**c**) Phase contrast-enhanced µCT slice with intracortical blood vessels visible (0.325 µm voxel size). (**d**) Identification of blood vessels (red) on histological section (Pentachrome). (**e**) Magnified cortical canal occupied by vasculature visible on CT data. (**f**) Magnified histological section with blood vessel visible in red colour (Giemsa staining). (**g**) Magnified cortical canal occupied by vasculature visible on CT data. (**h**) Magnified histological section with blood vessel visible in red colour (Pentachrome staining). (**i**) Magnified cortical canal occupied by vasculature visible on CT data. (**j**) Magnified histological section with blood vessel visible in red colour (Pentachrome staining). (**k**) Quantification of cortical canals filled with blood vessels in histological sections and CT slices (mean values and SD; *p* = 0.629).

Bone samples were scanned at a voxel size of 1.3 μm and 325 nm at two different X-ray propagation distances. Figure 1 shows the phase contrast-enhanced imaging ability to detect cortical vascular structures in bone. Figure 1a shows a schematic representation of an intracortical canal occupied by a blood vessel with image intensity profiles for both propagation distances (Fig. 1b,d). The observed differences in the intensity profiles make it possible to detect the soft tissue comprising the blood vessel at a phase-sensitive propagation distance (and not for standard X-ray tomography) (Fig. 1c,e). Larger vascular structures can be identified at a voxel size of 650 nm (Fig. 2a) but the smallest intracortical capillaries (~5 μm diameter and > 1 μm wall thickness) could only be detected at a voxel size of 325 nm as shown in Fig. 2c.

### Validation with histology

Microscopic analysis of calcified bone sections stained with either Pentachrome or Giemsa revealed the presence of blood vessels within intracortical canals demonstrating that the structures identified by phase contrast-enhanced tomography are blood vessels (Fig. 2b,d,f,h,j). Quantification of cortical canal occupancy by blood vessels was carried out on histological and CT data with high % of canal occupancy revealed by both techniques (86.91 ± 12.54 and 95.77 ± 1.55; Fig. 2k). No significant differences were found between the occupancy results provided by the two techniques (*p* = 0.629) when a non-parametric Mann-Whitley test was performed using GraphPad Prism version 6.0 for Windows (La Jolla, CA, USA).

### Vascular parameters

Computations for canal volume density, canal diameter and canal space occupied by the vascular structure were performed on CT data with mean value and standard deviation results shown in Table 1 for n = 4 datasets. % of canal volume was found to be 0.81 ± 0.12, the average canal diameter was 7.98 ± 0.70 microns and vascular space 85.45 ± 2.85% of the available canal space.

**Table 1:** Indices for 15 weeks old C57BL/6 female mice.

**Parameter**	**Mean**	**SD**
Canal Volume(%)	0.809	0.117
Canal Diameter(microns)	7.984	0.698
Canal Occupancy(%)	95.771	1.548
Vascular Space(%)	85.449	2.851
Number of Osteocyte Lacunae(# per mm3)	62.335 × 10^7^	0.719 × 10^7^

Indices are: canal volume normalised with total cortical volume, average canal diameter, canal occupancy by blood vessels, vascular volume normalised with cortical canal volume and osteocyte lacunae density normalised with cortical bone volume. Quantification performed in 4 X-ray tomography datasets, using the total scanned area for canal volume, canal diameter and osteocyte density indices and 60 sliced per dataset for canal occupancy and vascular space indices.

### Simultaneous visualisation of the cortical porosity comprising the calcified intracortical microstructure

**Figure 3:**
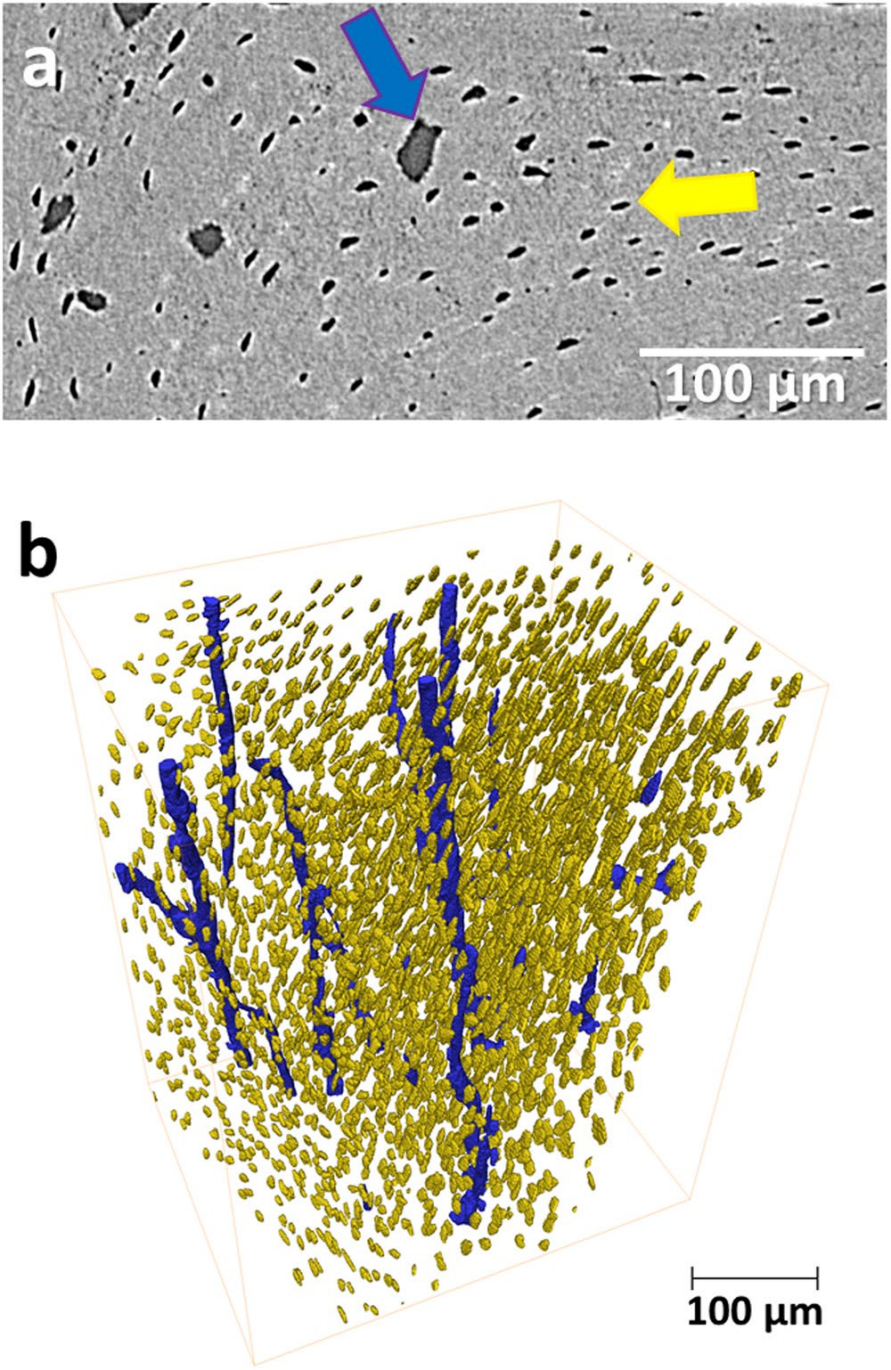
Cortical microstructure. Identification of intracortical canals (blue) and osteocyte lacunae (yellow) on micro-computed tomography (µCT) slice (**a**) and 3D rendering of intracortical canals (blue) and osteocyte lacunae (yellow) (**b**). Images extracted from 1.3 µm voxel size µCT dataset.

The cortical canal network and osteocyte lacunae were also assessed alongside with the soft tissue comprising the vasculature and cortical porosity extracted from standard X-ray data and classified into vascular canals and osteocyte lacunae (Fig. 3a,b). The assessment of the calcified cortical microstructure allowed the quantification of osteocyte lacunae density which was found to be 62.34 × 10^7^ ± 0.72 × 10^7^ lacunae per mm^3^ of mineralised tissue. Furthermore, the mineralised cortical geometry could be used to derive porosity measurements and mineralised tissue volume computations.

### 3D visualisation of intracortical blood vessels

**Figure 4:**
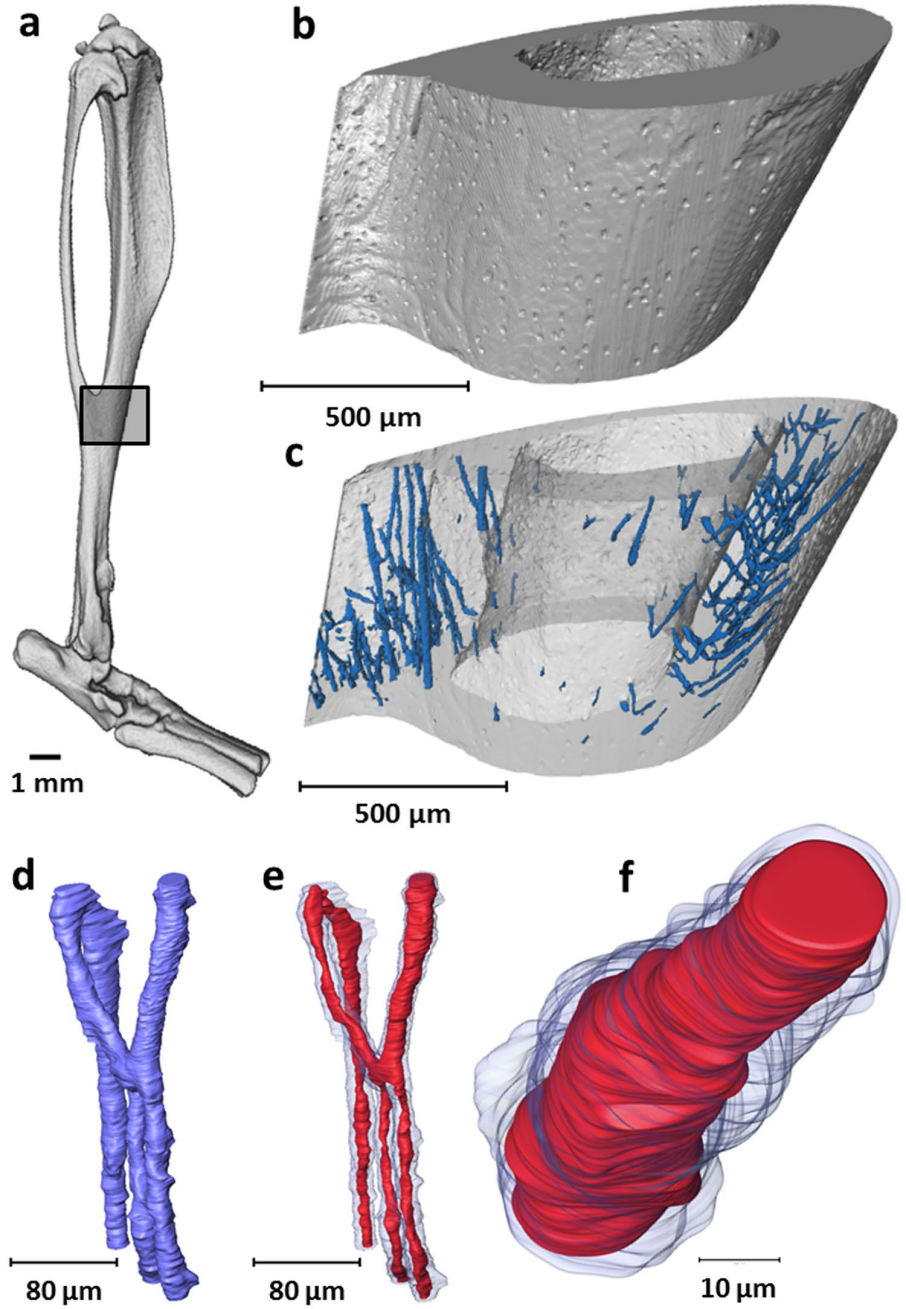
3D visualisation and extraction of intracortical blood vessel. (**a**) 3D rendering of murine tibia with identification of the tibiofibular junction. (**b**) 3D rendering of scanned tibiofibular junction region (1.3 µm voxel size) and (**c**) detection of intracortical canals (blue) as a negative imprint of the mineralised tissue (extracted from 1.3 µm voxel size dataset). (**d**) Magnified area of the 3D intracortical network (extracted from 0.325 µm voxel size dataset) and (**e**) detection of the soft tissue comprising blood vessels (red) within intracortical canals (blue) (extracted from 0.325 µm voxel size dataset). (**f**) Magnified segment of blood vessel (red) within intracortical canal (blue) (extracted from 0.325 µm voxel size dataset).

The SR CT images at the murine tibiofibular junction (Fig. 4a,b) were processed to extract the intracortical canal network (Fig. 4c). The extracted intracortical canal network (Fig. 4d) was then further explored and the soft tissue comprising the blood vessels segmented (Fig. 4e,f).

## Discussion

We have demonstrated that phase contrast-enhanced tomography allows imaging of the intracortical vasculature in unstained, unperfused and undecalcified murine bone at sufficient spatial resolution to identify the presence of individual blood vessels within the cortical canals and their size. Importantly, the mineralised cortical geometry including vascular canals and osteocyte lacunae, could also be imaged in parallel with the soft tissue which could contribute to bone porosity measurements, bone tissue volume computations and computational mechanics. Histological examination confirmed that the structures detected within the cortical canals are blood vessels, proving the ability of X-ray phase contrast-enhanced tomography to assess the cortical bone vasculature. The approach allows the quantification of vascular parameters such as vascular canal density, diameter, occupancy and vascular space. We have also provided numerical values for these parameters in the young adult murine tibiofibular junction that can now be used to evaluate age-related and disease-associated deviations.

In the past, visualisation of the bone vasculature has been problematic due to the nature of the skeletal tissue itself and efforts have been focussed on inferring the 3D structure of the vascular network within bone. Histology has been a gold standard for years used in both clinical diagnosis and biomedical research. However, the technique requires special sample preparation including embedding, sectioning (most frequently preceded by decalcification) and staining and is also destructive and two-dimensional.

Compared with histological examination, bone specimens can be evaluated in 3D and non-destructively using µCT; not requiring special sample preparation. Although conventional absorption-based µCT is not sensitive enough to provide sufficient contrast for soft tissues, such as the vasculature, it has been used to provide spatial clues for the location of the blood vessels that reside within the intracortical canal network^12–15^. The intracortical canal network can be extracted as a negative imprint of the calcified tissue from standard µCT images and several studies have done this in human^22–24^ and rodent^13,14,25,26^ bone using both lab-based and synchrotron X-ray sources. However, intracortical canals and blood vessels are not the same thing and from conventional absorption-based µCT data, it is not clear if specific “vascular” canals indeed contain blood vessels or not, unless additional histology has been performed. Further, we do not know if there are bone pathologies (or stages of pathologies) where blood vessels are lost from the canals, leaving the canals alone as a poor indicator of actual vessel structure. Therefore, the most significant limitation of conventional X-ray absorption-based µCT has been the inability to accurately assess both calcified and soft tissues simultaneously.

To overcome this limitation, investigations have been carried out in the past, perfusing the vascular circuit with a highly X-ray absorbing contrast agent to visualise the vasculature^12,27,28^. However there are some problems associated with these studies specifically that differences in the rheology of the perfused agent and the blood, it is very difficult to ensure that the agent has fully filled the vascular lumen (especially the smaller capillaries within the bone cortex), which results in disjoint vascular components and missing vascular segments as reported by Schneider *et al*.^12^. Most frequently, the contrast agent perfusion is used in conjunction with decalcified bone^27,28^ to allow image segmentation, which makes it impossible to assess the intracortical bone microstructure and the vascular morphology at the same time. Furthermore, when calcified samples are used, distinct segmentation of the vasculature and the mineralised tissue is problematic, not only because of the high X-ray absorption of the two components, but also due to the limited contrast between them.

There are of course limitations associated with this study as the use of phase contrast SR CT in bone. The main disadvantage of SR CT imaging is the availability of, and access to, the few SR sources worldwide (http://www.lightsources.org/regions). Allocation of scanning time is generally granted with proposals awarded on a competitive basis, with proposals submitted a half year in advance. Another limitation of our approach is that it has not been possible to observe vascular details at the submicron scale. Measurements of the vessel wall carried out on histological sections confirmed that the intracortical capillaries we are visualising generally have a wall thickness below the micrometre. At the current voxel size (325 nm) and resolution (2–3 times higher that voxel dimension), distinction of the two vessel surfaces (outer and luminal) is not possible. Thus, the single phase-related fringe we observe in our study corresponds to both outer and luminal surfaces of the microvessel; this is enough to determine the presence of the vascular structure within the canal and provide an indicator for its size. Increasing the spatial resolution will allow the detection of tube-like structures and access submicron vascular details which were beyond the scope of this work.

Synchrotron radiation dose for the described experiments is estimated to be below 6 MGy, and even though no damage leading to detrimental effects of the sample microstructure is expected^14,29,30^, we cannot consider this technique as non-invasive. In fact, the invasiveness of synchrotron radiation is a matter of definition according the application. For example, it has been reported that a similar radiation dose can alter the mechanical properties of bone^14,31,32^, which means that we need to take this into account when planning to perform mechanical tests. On the other hand, similar radiation dose has been proven to be compatible with immunohistochemical techniques with preservation of the epitopes^29^.

It is also important to highlight that even though local tomography is associated with image distortion and small perturbations in the image intensity, such artefacts are particularly a matter of concern in regions close to the boundary of the field of view and thus have been avoidable in our study. Furthermore, SR CT presents some advantages when compared with desktop X-ray sources; for instance, beam hardening effects are not present in SR CT since the radiation is monochromatic, in addition the fast acquisitions minimise artefacts related to sample movement or thermal stability.

To conclude, we have now shown that the bone vasculature and cortical bone microstructure in mice can be assessed simultaneously with no sample preparation. We hope our study can provide new opportunities to quantitate and better evaluate the role the vasculature plays in specific skeletal sites and different animal models. Visualisation of the 3D bone vascular network is a prerequisite for improved understanding of its role in the regulation of bone health and disease. The presented approach has the potential to link vascular abnormality to the pathogenesis of various skeletal conditions.

## Methods

### Animals

Mice were handled and surgical procedures conducted according to the guidelines of the Animals (Scientific Procedures) Act 1986. The murine bones required for this study have been obtained in compliance with EU Directive 2010/63/EU and have been approved by the Animal Welfare and Ethical Review Board of the University of Southampton, UK. 3-month-old (n = 4) and C57BL/6 female mice (Charles River Laboratories, Wilmington, Massachusetts, USA) were euthanised by cervical dislocation. The left and right tibiae were harvested, cleaned of soft tissue, fixed in 4% paraformaldehyde (pH 7.4) for 48 hours on a tube roller at 4 °C, and then preserved in 70% ethanol prior to µCT imaging and histology. Right tibiae were used for µCT imaging and contralateral left tibiae for histological analysis.

### Propagation-based phase contrast-enhanced imaging

Right tibiae were mechanically fixed with wax to prevent sample movement and the tibiofibular junctions were scanned using SR CT at the TOMCAT beamline of the Swiss Light Source at a voxel size of 1.3 μm and 325 nm (with the highest the spatial resolution needed to detect the presence and size of smallest intracortical capillaries) at two different sample-to-detector distances, (15 and 25 mm, for standard X-ray tomography and phase-sensitive imaging respectively,)^33,34^ (Fig. 1b,c). 1501 projections were acquired over a range of 180 degrees, at a photon energy of 21 keV, 18 ms exposure time per projection, which were corrected for ring artefacts due to potential scintillator defects and reconstructed using filtered backprojection. Figure 4a shows an entire murine tibia with the region scanned at the lower resolution highlighted and magnified in Fig. 4b. Scans were centred at the tibiofibular junction (maximum outer dimension of 1.5 mm). µCT datasets consisted of a stack of 2000 reconstructed µCT slices (Fig. 3a shows one µCT slice).

### Image processing and analysis

µCT datasets were processed and analysed using commercial software Avizo 9 (FEI, Oregon, USA) as well as freeware (ImageJ^35^). Image processing involved automatic segmentation of mineralised bone tissue and extraction of the intracortical canal network from the SR CT images at 1.3 μm voxel size and manual segmentation of soft tissue comprising blood vessels from the SR CT data acquired at 325 nm voxel size (extraction of the smallest intracortical capillaries was only possible with the highest resolution and manual segmentation of the images). Supplementary Fig. S1 summarises the image processing workflow adopted in this study to segment canals and osteocyte lacunae and supplementary Methods S1 details image processing operations. Parameters for the quantification were defined as: vascular canal density (%): volume of cortical canals in the CT dataset normalised with the total cortical tissue volume; average canal diameter (microns): mean diameter dimension of the canals in the CT dataset; osteocyte lacunar density (number per mm^3^ of mineralised volume): number of osteocyte lacunae in the CT dataset per unit of mineralised cortical bone; canal occupancy (%): percentage or canal structures that are filled with a blood vessel (note that this parameter has been quantified in both CT and histological data, for the CT data 60 reconstructed CT slices per dataset were inspected); and vascular space (%): area of the vascular structure normalised with the total canal area (parameter computed using 60 CT slices per dataset),

### Histology

Contralateral left tibiae were dissected free of soft tissues. Dehydration was carried out by immersing tissue in a series of ethanol solutions of increasing concentrations until 100% was reached and the tissue could be infiltrated with the embedding material. Bone cutters (Fine Science Tools, California, USA) were used to section just above the tibiofibular junction (region of interest) and to cut the upper region of the tibia off. Following dehydration, bones were soaked for 24 hours in polymethylmethacrylate (PMMA) at 4 °C. PMMA blocks were ground down using a grinder-polisher (MetaServ 250; Buehler, Illinois, USA) so that the cut surface was flat. 10-µm sections were cut out from the proximal end to trim the bone using a manual rotary microtome (Leica Biosystems, Wetzlar, Germany) until the tibiofibular junction was reached. 5-µm sections were cut and one drop of 80% isopropanol solution added to each section to prevent folding. Sections were pressed in an incubator at 37 °C overnight to dry the tissue and stained following either Pentachrome or Giemsa protocols to confirm the presence of blood vessels.

## Electronic supplementary material

Supplementary Methods and Supplementary Figure
