# Supplementary material for: Simultaneous visualisation of calcified bone microstructure and intracortical vasculature using synchrotron X-ray phase contrast-enhanced tomography
Source: Sci Rep. 2017 Oct 16;7:13289. doi: 10.1038/s41598-017-13632-5 (PMC5643345; doi:10.1038/s41598-017-13632-5)

Simultaneous visualisation of calcified bone microstructure and intracortical vasculature using synchrotron X-ray phase contrast-enhanced tomography

Juan A Núñez[1,2*], Alice Goring[1], Eric Hesse[3,4] Philipp J Thurner[2,5†], Philipp Schneider[2†] and Claire E Clarkin[1]

[1]Biological Sciences, Faculty of Natural and Environmental Sciences, University of Southampton, SO17 1BJ, UK

[2]Bioengineering Sciences Research Group, Faculty of Engineering and the Environment, University of Southampton, SO17 1BJ, UK

[3]Heisenberg-Group for Molecular Skeletal Biology, Department of Trauma, Hand and Reconstructive Surgery, University Medical Center Hamburg-Eppendorf, Martinistrasse 52, D-20246 Hamburg, Germany.

[4]Department of Anatomy and Cell Biology, Indiana University School of Medicine, Indianapolis, IN, USA

[5]Institute of Lightweight Design and Structural Biomechanics, Vienna University of Technology, 1060 Vienna, Austria

Supplementary Information

- **Supplementary Methods S1. Image processing analysis**
- **Supplementary Fig. S1. Image processing workflow**

Supplementary Methods S1. Image processing analysis

SR CT datasets were processed and analysed using commercial software Avizo 9 (FEI, Oregon, USA) as well as freeware (ImageJ) to segment the mineralised bone tissue and extract the bone porosity or intracortical microstructure (i.e., vascular canals and the osteocyte lacunae at the present spatial resolution). Supplementary Fig. S1 summarises the image processing workflow adopted in this study.

The first step was the segmentation of the mineralised tissue. The bimodal 3D image histogram was thresholded using the intermodes global method in ImageJ. Global thresholding is an image segmentation method that replaces a pixel in a greyscale image with a black pixel if the image intensity is less than some fixed constant (the threshold), or a white pixel if the image intensity is greater than that constant. The intermodes global thresholding identifies the values of two histogram peaks and uses the midpoint as threshold. The output of the thresholding operation is a binary image (image composed of two intensity values only, namely object and background).

After the thresholding operation, to improve accuracy in the segmentation, a series of operation were performed to remove some clusters of pixels that should be part of the background of the image and have been incorrectly identified as mineralised tissue. This second step, namely cleaning, starts with a 3D opening operation followed by a component labelling with individual components detected sorted by volume in Avizo 9. The labelled image is then sieved to keep only the largest component (cortical bone) in Avizo 9 and all the cluster of pixels that were incorrectly included as mineralised tissue removed.

The following group of operations were performed to generate an image of the solid cortex (mask). A 3D closing operation (Avizo 9) incorporates the osteocyte lacunae that have been detected as part of the background because of their proximity to the bone surfaces. Subsequently, cortical pores are removed with an operation that fills all the holes smaller than a fixed value (determined by the size

of the medullary cavity) in Avizo 9. A 3D erosion operation (Avizo 9) thins the solid cortical image to ensure that the final mask image is within the actual mineralised cortex.

In order to extract cortical porosity, the thresholded data (binary image produced in the first step of the process) is then inverted and combined with the mask in an “and” logical operation (Avizo 9) resulting in a dataset containing the pores. A 3D opening operation is applied (Avizo 9) to separate clusters of pores. The separated pores are sorted via component labelling and a bounding box is computed for each pore. The maximum dimension of each bounding box was used to classify the particles into noise, osteocyte lacunae and canals using maximum dimension criteria.

Supplementary Fig. S1

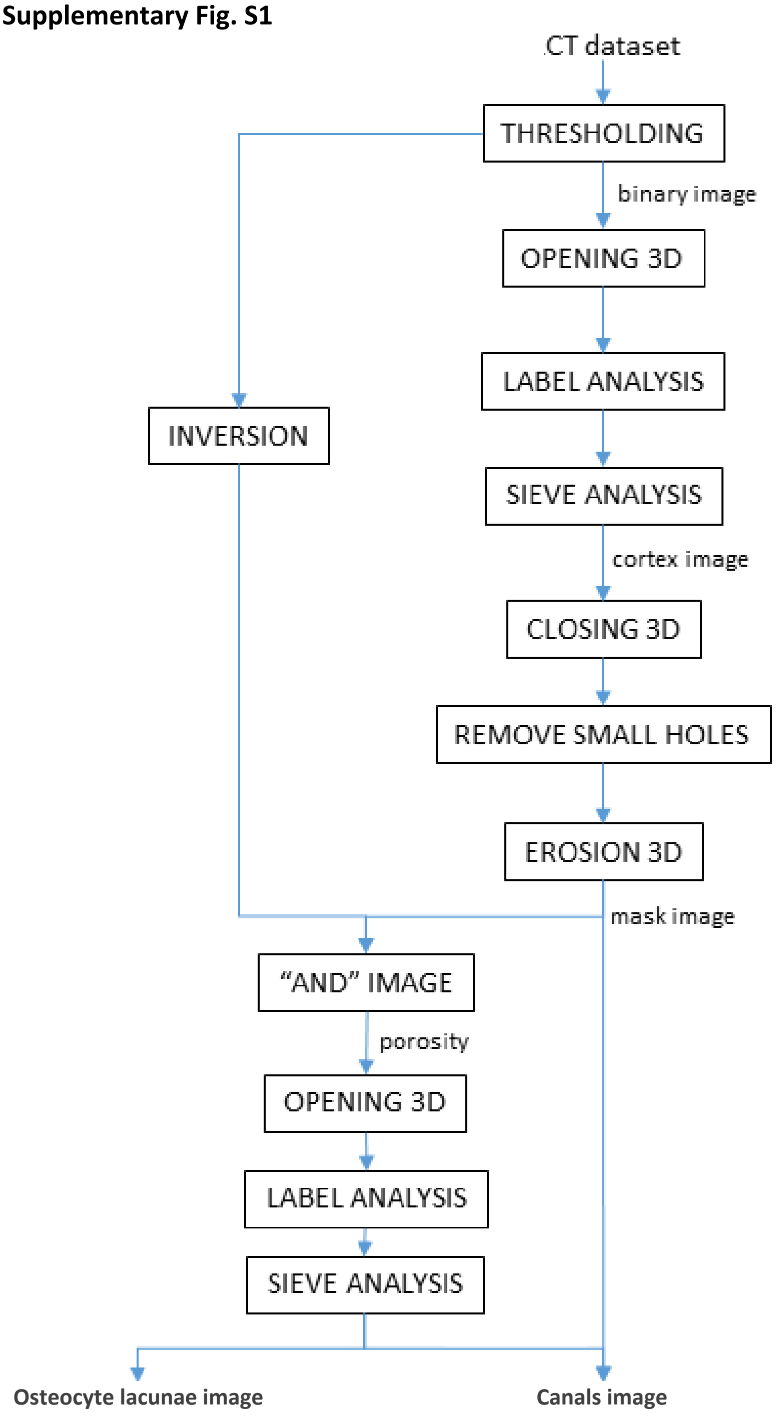

Supplement: Supplementary file 1 — Supplementary Methods and Supplementary Figure [file 41598_2017_13632_MOESM1_ESM.pdf]
